# A difficult situation – balancing critical anticoagulation versus the risk of permanent neurologic deficit: a case report

**DOI:** 10.1186/s13256-018-1688-x

**Published:** 2018-06-22

**Authors:** Girard Cua, Neal Holland, Ashleigh Wright

**Affiliations:** University of Florida College of Medicine, 1600 SW Archer Rd, Gainesville, FL 32610 USA

**Keywords:** Pulmonary embolism, Retroperitoneal hematoma, Anticoagulation, Neurologic deficit

## Abstract

**Background:**

Anticoagulation is the mainstay of treatment for pulmonary embolism. However, if bleeding unfortunately occurs, the risks and benefits of anticoagulation present a challenge. Management of one hemorrhagic complication, retroperitoneal hematoma, is rare, difficult, and controversial.

**Case presentation:**

A 73-year-old white man presented with left lower extremity swelling and dyspnea. He was tachycardic, hypertensive, and demonstrated poor oxygen saturation of 81% on ambient air. A computed tomography angiogram revealed a saddle pulmonary embolus. Tissue plasminogen activator was administered and he was started on a heparin infusion. He was eventually transitioned to enoxaparin. On the day of discharge, however, he had sudden onset of right leg numbness and weakness below his hip. A computed tomography of his head was not concerning for stroke, and neurology was consulted. Neurology was concerned for spinal cord infarction versus hematoma and recommended magnetic resonance imaging of his thoracic and lumbar spine. The magnetic resonance imaging revealed a left psoas hematoma. A computed tomography scan of his pelvis also showed a right psoas and iliacus hematoma. He was transitioned to a low intensity heparin infusion. The following day his left leg exhibited similar symptoms. There was concern of progressive and irreversible nerve damage due to compression if the hematomas were not drained. Interventional radiology was consulted for drainage. The heparin infusion was paused, drainage was performed, and the heparin infusion was reinitiated 6 hours following the procedure by interventional radiology. His blood counts and neurologic examination stabilized and eventually improved. He was discharged home on a novel anticoagulant.

**Conclusions:**

Management of a retroperitoneal hematoma can commence with recognition of the warning signs of bleeding and neurological impairment, and consulting the appropriate services in case the need for intervention arises. A conservative approach of volume resuscitation and blood transfusion can be used initially, with the need for pausing or reversing anticoagulation being assessed on an individual basis with expert consultation. If intervention becomes necessary, other interventional radiology-based modalities can be used to identify and stop the bleeding source, and interventional radiology-guided drainage can be performed to decrease the hematoma burden and relieve neurological symptoms.

## Background

The management of retroperitoneal hematomas in the setting of critical anticoagulation is rare, difficult, and controversial. There exists the need to strike a delicate balance. Management can commence with early recognition of the warning signs of occult bleeding and neurological impairment, and consulting the appropriate services in case an imminent need for intervention arises. A conservative approach of volume resuscitation and blood transfusion can be used initially, with the need for pausing or reversing anticoagulation being assessed on a case-by-case basis with expert consultation. If intervention becomes necessary, modalities such as transarterial embolization (TAE) can be used to identify and stop the bleeding source, and interventional radiology (IR)-guided drainage can be performed to decrease the hematoma burden and relieve neurological symptoms. Finally, as with all clinically complex cases, patient awareness and informed consent play a major role in how the plan is implemented. We describe a case in which a patient was admitted to our hospital after sustaining a saddle pulmonary embolus (PE), received appropriate anticoagulation, and subsequently developed bilateral retroperitoneal hematomas as a complication of therapy. This case highlights an unexpected complication of a commonly treated condition, as retroperitoneal hematomas in the setting of anticoagulation are rarely reported in the literature [1]. In addition, our publication review yielded very few case reports in which retroperitoneal hematomas occurred as a complication of pulmonary embolism treatment specifically.

## Case presentation

**Fig. 1:**
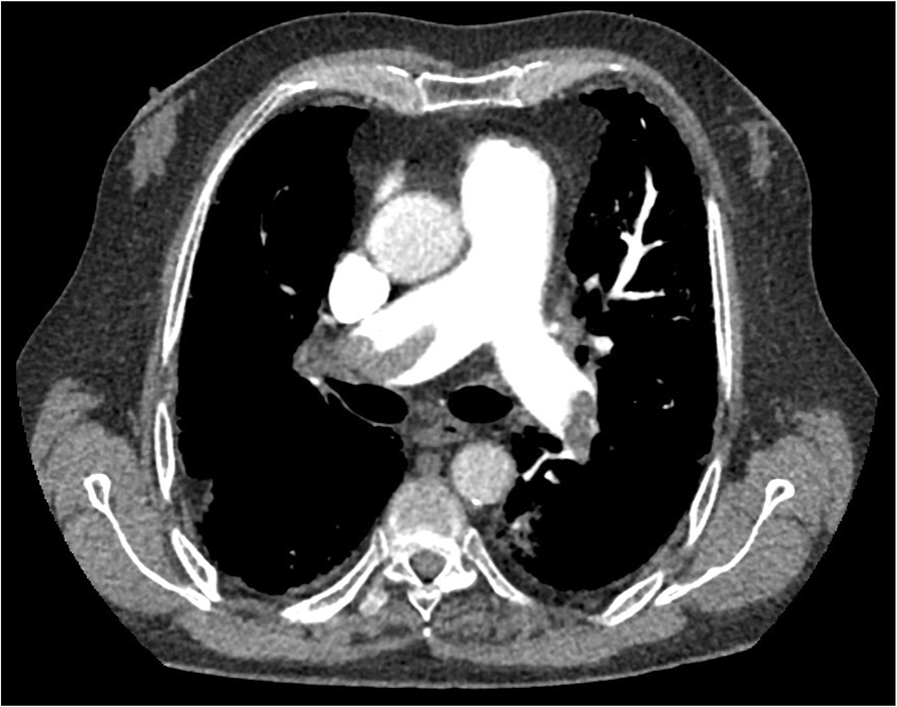
Computed tomography showing the saddle pulmonary embolus

A 73-year-old white man presented to our emergency department with a 3-day history of left lower extremity swelling and acute-onset shortness of breath. On evaluation, he was tachycardic with a pulse of 113, hypertensive with a systolic blood pressure of 130-170 mmHg, and demonstrated poor oxygen saturation of 81% on room air. He was supported with continuous positive airway pressure (CPAP) and supplemental oxygen while a computed tomography angiogram (CTA) was obtained, which revealed a saddle PE (Fig. 1).

Tissue plasminogen activator (tPA) was administered and he was started on a heparin infusion and admitted to our intensive care unit (ICU) for management. He remained on the heparin infusion for 3 days, during which he continuously improved and was eventually weaned to 3 L oxygen via nasal cannula. On hospital day 2, he was transferred to intermediate level of care. Per hematology recommendations, he would have to be on indefinite anticoagulation due to the massive PE he had sustained, the source of which was a left lower extremity popliteal deep vein thrombosis (DVT). The plan was to transition him from the heparin infusion to enoxaparin twice per day with hematology follow-up in 1 month.

### Clinical findings

On the day of discharge, however, he had sudden onset of right leg numbness and weakness below the level of his hip. He had previously been working with physical therapy and had been able to walk 200 feet with the assistance of a walker during each session. A physical examination revealed decreased sensation to light touch, 2/5 strength in right hip flexion and right knee extension and flexion, and loss of right patellar reflex. Left leg physical examination was normal at that time.

### Diagnostic assessment

**Fig. 2:**
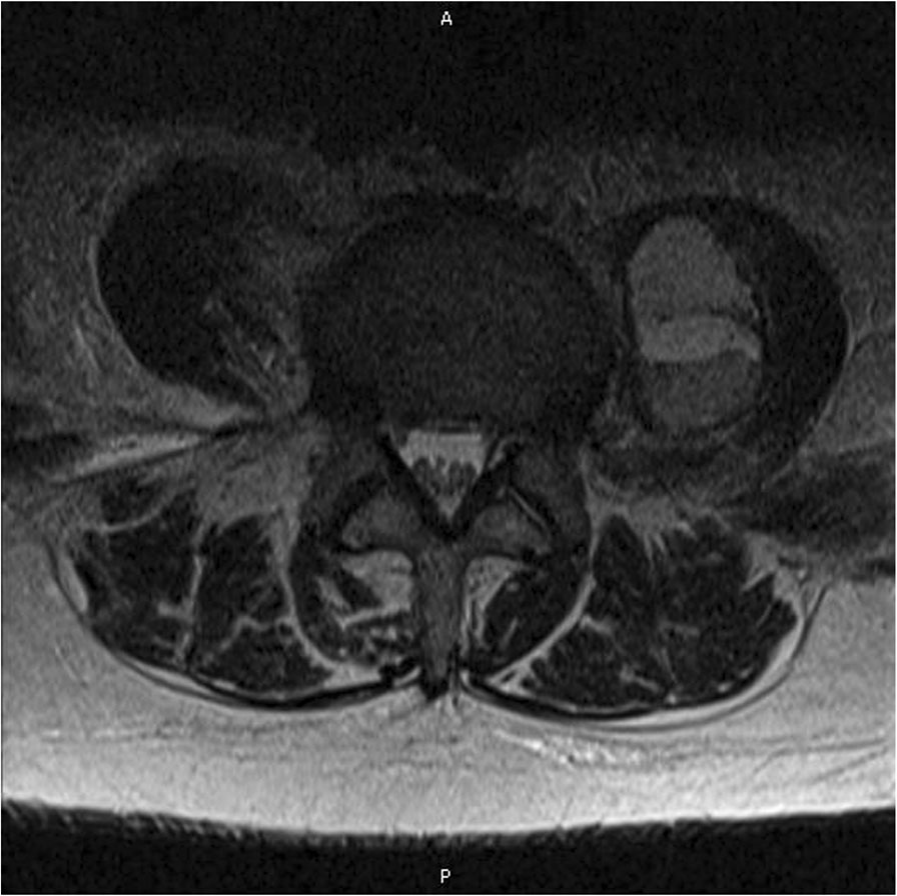
Magnetic resonance imaging showing the left psoas hematoma

An emergent head computed tomography (CT) scan was ordered due to concern for a possible stroke, and neurology was consulted. The head CT was negative for infarction or hemorrhage. Neurology was concerned for spinal cord infarction versus hematoma and recommended emergent magnetic resonance imaging (MRI) of his thoracic and lumbar spine. The MRI revealed a left psoas hematoma (Fig. 2). A CT of his pelvis performed the same day also showed a right psoas and iliacus hematoma. Due to these findings, hematology recommended discontinuing enoxaparin and reverting to a low intensity heparin infusion, as well as placement of an inferior vena cava (IVC) filter. The following day his left leg began exhibiting the same symptoms as his right leg. There was concern regarding the risk of progressive and irreversible nerve damage due to compression if the hematomas were not promptly drained. IR was consulted and advised that if the drainage were to occur while our patient was on anticoagulation, the risk of rebleeding into the retroperitoneum would be high and potentially nullify any benefit from drainage. Our patient would also be at risk of hemodynamic instability if the current hematomas were acting as tamponades against further bleeding. An additional complicating factor was the risk of further thrombosis due to the presenting saddle PE. Hematology was consulted for recommendations on pausing anticoagulation, but they were hesitant to offer a timeframe as there was no established safe period to enable this type of procedure to take place. Eventually, a window period of pausing the heparin infusion for 3 hours pre-procedure and up to 6 hours post-procedure was decided upon in the event that our patient agreed to have the drainage performed.

Throughout this sequence of events, our patient and his wife were aware of the plans and considerations on how to proceed. They were informed of the recommendations and concerns made by neurology, hematology, and IR, as well as the risks and benefits of intervention versus non-intervention.

### Therapeutic intervention

**Fig. 3:**
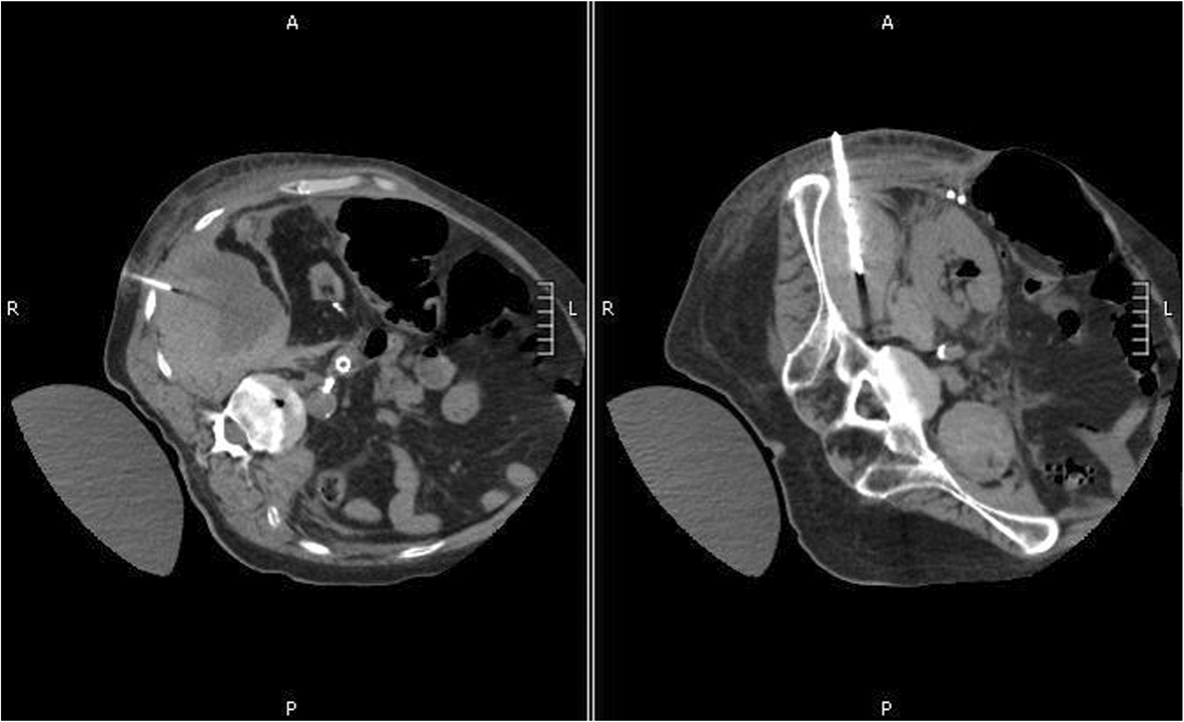
Computed tomography showing interventional radiology-guided drainage of the right retroperitoneal hematoma

After speaking with his wife, our patient decided to undergo the procedure. The low intensity heparin infusion was stopped 3 hours beforehand and the IR team then performed drainage of the right retroperitoneal hematoma, placing two pigtail catheters in our patient’s right flank (Fig. 3). In total, the hematoma was drained of 215 milliliters of blood, 10 milliliters of which were drained during the procedure itself. The left psoas hematoma was not found to be amenable to drainage.

### Follow-up and outcomes

**Fig. 4:**
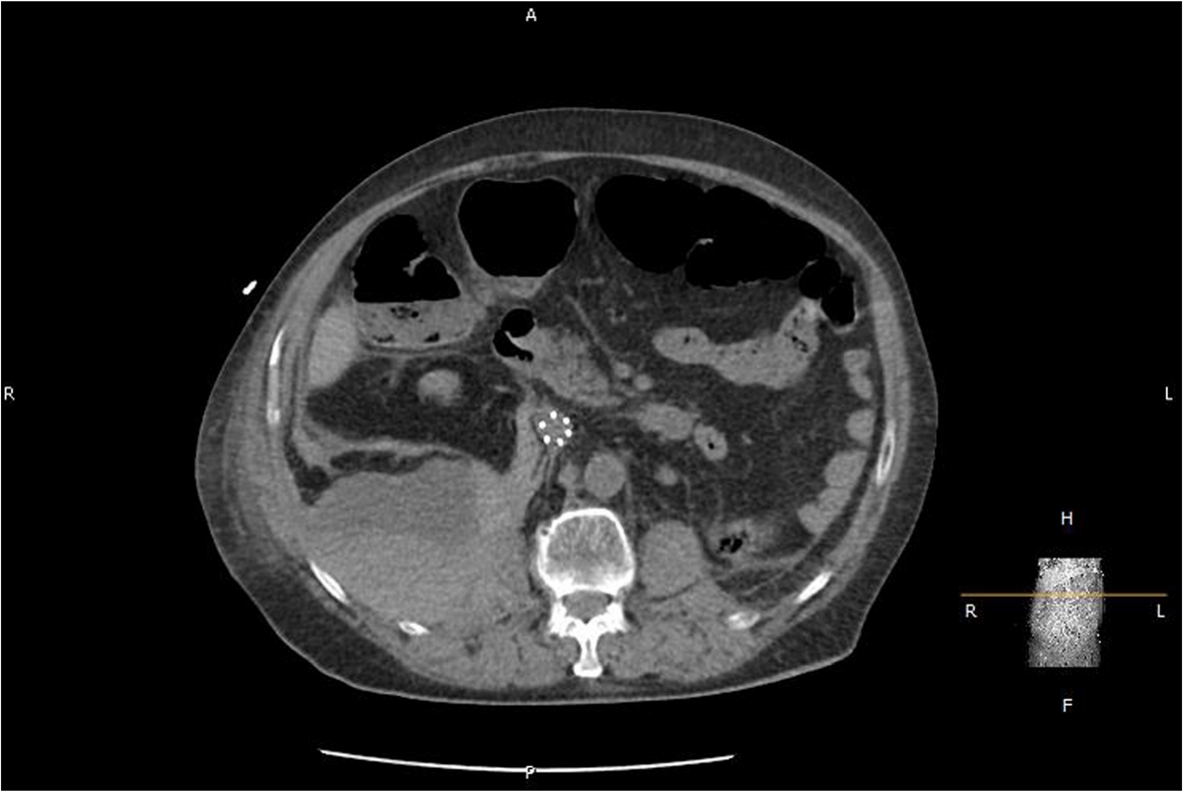
Repeat abdomen and pelvis computed tomography performed on hospital day 10. The implanted inferior vena cava filter can also be seen

Our patient tolerated the procedure well, and the heparin infusion was restarted 6 hours after it was completed. A repeat neurological examination demonstrated improved lower extremity strength bilaterally as well as the return of sensation to light touch. Hip flexion improved to 3/5 bilaterally, and knee flexion and extension improved to 4/5 bilaterally. Deep tendon reflexes remained absent. Four days later, the pigtail catheters were removed. His recovery was complicated by anemia requiring blood transfusions totaling 4 units of packed red blood cells (PRBC). Other sources of potential bleeding were evaluated and not found. A repeat CT on hospital day 10 (Fig. 4) showed a stable right-sided hematoma, and our patient did not experience any further neurologic deficits. He was transitioned again from the heparin infusion to enoxaparin after 3 more days. His hemoglobin and hematocrit remained stable. During this time, he worked with physical therapy, who recommended discharge to a skilled nursing facility where his strength began to improve somewhat. Follow-up was scheduled with neurology and hematology. On hospital day 18, he was safely discharged.

## Discussion

The complexity of this case far exceeded initial expectations, bringing with it several valuable learning points. Parenteral anticoagulation should be given to patients with confirmed acute PE. The risk of major bleeding with anticoagulation therapy is less than 3% [2]. Bleeding is classified as “major” if it is intracranial, retroperitoneal, leads to hospitalization, the need for blood transfusion, or death [3]. In terms of retroperitoneal hematomas, the occurrence rate approaches 0.5–1% in patients on systemic anticoagulation [3, 4], with retroperitoneal bleeds almost exclusively seen in states of anticoagulation, coagulopathies, and hemodialysis [5]. In general, risk factors for bleeding are: age > 65 years, previous bleeding, thrombocytopenia, antiplatelet therapy, poor anticoagulant control, recent surgery, frequent falls, reduced functional capacity, previous stroke, diabetes, anemia, cancer, renal failure, liver failure, and alcohol abuse [2]. In transitioning from unfractionated heparin to low molecular weight heparin, the major bleeding risk appears to decrease from 2% to 1.2%, respectively [2]. In our case, the patient’s risk factors included his age, recent administration of tPA, and ongoing anticoagulation therapy with enoxaparin due to his PE.

Patients with retroperitoneal hemorrhage can present in a variety of ways. One third of patients will present with Lenk’s triad: severe flank pain, hemodynamic shock, and palpable mass [6]. The remainder of patients can have symptoms consisting of: nausea, vomiting, abdominal distension, intestinal obstruction, hypovolemia, anemia, limb swelling, paresthesia, femoral nerve compression causing paralysis of a lower limb, muscle weakness, reduced knee or thigh reflex, increased intra-abdominal pressure, and/or abdominal compartment syndrome [7]. It is therefore important to be aware of these potential complications when starting patients on anticoagulation. Proposed mechanisms that may precipitate retroperitoneal hemorrhage are: forceful muscle strain, diffuse small vessel arteriosclerosis, heparin-induced immune microangiopathy, and unrecognized minor trauma [6].

Treatment of spontaneous retroperitoneal hematomas in the setting of anticoagulation is difficult. There is lack of Level I evidence for the best management plans, with most evidence based on case reports [8]. Upon review of selected case reports, there always exists the risk of stopping anticoagulation versus continued bleeding. The general pattern found in the case reports initially begins conservatively, with increasing invasiveness as chronicity and severity increases. In addition, CT imaging of the abdomen and pelvis should be performed in order to document the type, site, and extent of the suspected hematoma [8]. In a case report by Gurbuz *et al.*, a 41-year-old patient developed femoral nerve palsy due to a retroperitoneal hematoma caused by recent addition of aspirin to his already existing warfarin therapy for a mitral valve prosthesis [3]. His international normalized ratio (INR) was supratherapeutic at 4.1. He was successfully treated by reversing warfarin with 10 mg of vitamin K and 2 units of fresh frozen plasma (FFP), along with right leg elevation above his heart. His neurologic function recovered and the hematoma remained stable, gradually reabsorbing by 3-week follow-up. Our case differs from theirs as the bleed appears to have been caused by a supratherapeutic INR as well as addition of an antiplatelet medication.

Another modality which could have been considered in our case was the use of TAE in order to target and stop the bleeding source. A case report by Wada *et al.* described bilateral iliopsoas hematomas that developed in an 85-year-old patient undergoing anticoagulation for transient ischemic attack (TIA) [9]. Her symptoms consisted of: pain, weakness, and decreased sensation in the groin bilaterally [9]. She underwent aortography and, although no contrast extravasation was seen, underwent TAE in the third and fourth lumbar artery trunk due to the suspected origin at that location. Her symptoms progressively improved by the third week. In another case, Won *et al.* employed both TAE as well as surgical decompression of a spontaneous retroperitoneal hematoma in a 48-year-old woman undergoing warfarin and aspirin anticoagulation for an artificial aortic valve [6]. She presented with hemodynamic instability and an INR of 4.6. Angiography showed right internal iliac artery extravasation, which was embolized with TAE. However, her neurologic symptoms continued to worsen with the development of anuria and abdominal compartment syndrome that necessitated exploratory laparotomy.

Other therapeutic options for our patient included a TAE before the IR-guided drainage in order to stop the bleeding source. This would have reassured us that a repeat bleed would be less likely to occur. However, while TAE may stop the source of the bleed, it does not reduce the hematoma burden on the nerves and, therefore, would not have reversed the neuropathy. In addition, our patient had already developed an acute kidney injury (AKI) and did have a history of kidney transplant in 2006. A contrasted study at that point in time would not have been advisable. There is also the risk of failing to identify the bleed source even with angiography, as was seen in the case presented by Wada *et al. [*9*].*

In all reported cases where intervention was necessary, anticoagulation was either discontinued or reversed, introducing the risk of thrombosis. However, none of the cases mentioned thrombosis as a complication of their treatment. With our patient, we did not feel comfortable reversing or discontinuing the anticoagulation as, in our case, the patient was recovering from an acute thrombotic event. It would be difficult to predict the risk of hematoma recurrence after drainage, as retroperitoneal hematomas are rare. However, it is logical that, if the source of bleeding were contained with a procedure such as TAE, the risk of rebleeding would be decreased.

The management of retroperitoneal hematomas in the setting of critical anticoagulation is rare, difficult, and controversial. There exists the need to strike a delicate balance. Management can commence with early recognition of the warning signs of occult bleeding and neurological impairment, and consulting the appropriate services in case an imminent need for intervention arises. A conservative approach of volume resuscitation and blood transfusion can be used initially, with the need for pausing or reversing anticoagulation being assessed on a case-by-case basis with expert consultation. If intervention becomes necessary, modalities such as TAE can be used to identify and stop the bleeding source, and IR-guided drainage can be performed to decrease the hematoma burden and relieve neurological symptoms. Finally, as with all clinically complex cases, patient awareness and informed consent play a major role in how the plan is implemented.

## Conclusions

1. Patients with confirmed PE should be given parenteral anticoagulation, with the risk of major bleeding being less than 3%. There are several risk factors that predispose patients to bleeding.
2. Spontaneous retroperitoneal hematoma is a rare clinical entity seen almost exclusively during anticoagulation, coagulopathies, or dialysis. Early recognition of signs of bleeding is important and advised in the management of patients in this population because it is a serious and potentially lethal complication.
3. Intervention has generally consisted of conservative measures first, then progressing to more invasive modalities as the chronicity or severity of the patient’s condition manifests itself. The need for continuing anticoagulation should be reassessed, and risks and benefits should be explained to the patient before proceeding with any plan of action.

## Abbreviations

AKI: Acute kidney injury
CPAP: Continuous positive airway pressure
CT: Computed tomography
CTA: Computed tomography angiogram
DVT: Deep vein thrombosis
FFP: Fresh frozen plasma
ICU: Intensive care unit
INR: International normalized ratio
IR: Interventional radiology
IVC: Inferior vena cava
MRI: Magnetic resonance Imaging
PE: Pulmonary embolus
PRBC: Packed red blood cells
TAE: Transarterial embolization
TIA: Transient ischemic attack
tPA: Tissue plasminogen activator
